# Taringa Whakarongo: Kaumātua & whānau experiences of hearing loss & hearing healthcare in Tāmaki Makaurau

**DOI:** 10.1080/03036758.2024.2424793

**Published:** 2024-12-12

**Authors:** Alehandrea Raiha Manuel, Elana Curtis, Grant Searchfield

**Affiliations:** a School of Population Health, Faculty of Medical and Health Sciences, Waipapa Taumata Rau The University of Auckland, Tāmaki Makaurau Auckland, Aotearoa New Zealand; b Te Kupenga Hauora Māori, Faculty of Medical and Health Sciences, Waipapa Taumata Rau The University of Auckland, Tāmaki Makaurau Auckland, Aotearoa New Zealand; c Section of Audiology, Faculty of Medical and Health Sciences, Waipapa Taumata Rau The University of Auckland, Tāmaki Makaurau Auckland, Aotearoa New Zealand

**Keywords:** Hearing healthcare, equity, Kaupapa Māori research, Kaumātua, Whānau, Hauora, hearing health, hearing loss, qualitative research, audiology

## Abstract

The ‘Taringa Whakarongo’ [TW] project presents the first narratives on hearing loss and hearing healthcare [HHC] among kaumātua and whānau. The purpose behind the project was to explore kaumātua and their whānau lived experiences of hearing loss and HHC. Hearing health care professionals’ and Māori health professionals’ perspectives were also examined. Through a Kaupapa Māori lens and reflexive thematic analysis, the sense of hearing was identified as a taonga among kaumātua. However, colonisation, societal stigma of hearing loss, and barriers to accessing whānau-centred HHC and hearing technology continue to impact kaumātua with hearing difficulties and their whānau. Hard-of-hearing kaumātua can thrive in their communities when they and their whānau are supported. Highlighted in this article are key recommendations for the Crown and health sector based on research partner narratives including valuing and prioritising whānau-centered care; supporting whakawhanaungatanga, Māori HHC leadership and culturally safe care; continuing Kaupapa Māori research endeavours and adopting an Indigenous rights-based approach to hearing health.

**Glossary of kupu Māori (Māori words): Hauora Māori**: Māori health; **Ihirangaranga**: Healing sound vibrations; **Iwi**: Tribe/s; **Kanohi ki te kanohi**: Face-to-face; **Karakia**: Prayer/blessing; **Karanga**: Ceremonial call or summoning; **Kaumātua**: Māori elder/s; **Kaupapa**: Main purpose of the encounter or collective vision; **Kaupapa Māori**: Embedded within Māori practices, values, beliefs, it is a by Māori, for Māori, with Māori, philosophical and relational way of doing, connecting, being, and thinking; **Kaupapa whānau**: Members with a common purpose or disabling experience who provide supporting and nurturing roles that traditional whānau provide; **Koha**: Gift; **Kōrero**: speak/discussion; **Mana**: Power; **Mana motuhake**: Independence, autonomy; **Manaakitanga**: The act of support and showing respect; **Māori**: The Indigenous people of Aotearoa New Zealand also refers to normal/ordinary; **Māori-ki-te-Māori**: Māori to Māori; **Marae**: Māori communal and sacred meeting grounds where Māori values and philosophy are reaffirmed; **Mātauranga Māori**: Māori body of knowledge, epistemology, and worldview; **Me rongo i te whanaungatanga**: Feeling connected; **Mihimihi**: Greeting; **Mokopuna**: Grandchildren; **Paepae**: Place on the marae where orators sit, stand, and deliver their speeches; **Rōpū**: Group/s; **Taha hinengaro**: Mental and emotional wellbeing; **Tāmaki Makaurau**: Auckland region, Tāmaki-desired-by-many or Tāmaki of 100 lovers; **Tamariki Māori**: Māori child/ren; **tanga**: Being in the state of; **Tāngata whaikaha**: People with lived experiences of disability; **Tāngata whaikaha kaumātua**: Kaumātua with lived experiences of disability; **Tangata whenua**: People of the land; **Taonga**: a treasure to be valued and preserved; **Te Ao Māori**: The Māori world; **Te reo Māori**: The Māori language; **Te Taiao**: The natural world; **Te Tiriti o Waitangi (Te Tiriti)**: Te reo Māori version of a fundamental binding agreement in New Zealand between Māori and the British Crown; **Tino rangatiratanga**: Sovereignty, self-determination, sovereignty; **Waiata**: songs; **Whaikōrero**: Formal speech; **Whakamā**: Shy and ashamed; **Whakapapa**: Genealogical relation to all things living and non-living; **Whakapapa whānau**: Members genealogically connected by common ancestors; **Whakarongo**: Listen; **Whakawātea**: Reflections and clearing the pathway forward; **Whakawhanaungatanga**: The process of establishing and maintaining relationships; **Whānau**: Immediate and extended family network; **Whanaunga**: Relation; **Whenua**: Lands.

## Introduction

The proportion of older Māori (aged 65 years and over) in Aotearoa New Zealand is projected to double from 6% in 2018 to 12% by 2043 (Stats 2022). The shift in age demographics will significantly impact Māori elders (kaumātua) with unassisted hearing loss, with extensive research demonstrating that older adults who experience unaddressed hearing loss are vulnerable to a wide cascade of conditions (Lin et al. 2013; Gurgel et al. 2014; Davis et al. 2016; Fortunato et al. 2016; Taljaard et al. 2016; Livingston et al. 2017; Loughrey et al. 2018; Manuel et al. 2021). This is a matter of concern, with Māori self-reporting higher rates of disabling hearing loss despite lower access to assistive technology than non-Māori (Greville 2005; Thorne et al. 2008; Office for Disability Issues and Stats NZ 2010).

Evidence suggests that many kaumātua retain unique and positive perspectives of ageing intertwined with cultural values, their societal roles and contributions, sharing of knowledge, and their connections with whānau (immediate and extended family network) (Durie et al. 1997; Dyall et al. 2011; Butcher and Breheny 2016). More timely and accessible hearing healthcare (HHC) interventions could assist kaumātua to continue their intergenerational responsibilities and maintain quality relationships with their whānau and communities.

This article presents the findings of a Māori-led Kaupapa Māori (KM) project entitled ‘Taringa Whakarongo’ (TW) (Manuel 2022). The study aimed to explore kaumātua and their whānau lived experiences and perspectives of hearing loss and HHC in Tāmaki Makaurau (Auckland region). The study also captured and analysed hearing healthcare professionals’ (HHCPs) and Māori health professionals’ (MHPs) perspectives on hearing loss and HHC provision for kaumātua and whānau.

### Ethics

Ethics approval for this research was obtained from Auckland Health Regional Ethics Committee (Reference number: 000100). The Tōmaiora Māori Health Research Group (2015) guidelines, alongside a Kaupapa Māori (KM) approach supported a critical and ethical research approach overall.

## Methodology

### Kaupapa Māori research

TW takes a decolonial, transformative, intersectional approach through a KM paradigm. KM theory has evolved as a form of resistance of Māori communities, whose lived experiences, values, and history have been dominated and marginalised through Western research paradigms (Bishop 1999; Smith 2012). Embedding KM theory, methodology, and praxis in the TW study validates and highlights Māori research partners as experts, storytellers, and mātauranga Māori (Māori knowledge) holders in their experiences of hearing loss and HHC. The research team (ARM, EC, GS) ensured the following KM principles grounded KM praxis in TW: be beneficial to Māori and under Māori control; informed by mātauranga Māori; be accepting of diverse Māori realities; reject victim-blaming cultural-deficit theories; align with a structural determinants approach to critique issues of power, privilege, and racism; support a decolonising approach that is transformative in praxis for Māori (Curtis 2016). The team were also reflexive of their positioning within a KM approach to TW and played a pivotal role in balancing and mediating power dynamics, informing processes of the study, interpretation and write up of kaumātua and whānau voices. An advisory team that represented community and health professional voices provided guidance on the best way to deliver a transformative KM project that was mana (power) enhancing for all research partners.

### Qualitative methods

Qualitative methods included whānau interviews and focus groups. These methods appreciate Māori oral histories and traditions, and provide access to understanding historical and contemporary realities of hard-of-hearing Māori, their whānau, health professionals and the HHC system.

### Recruitment and data collection

#### Whānau interviews

Whānau interviews included kaumātua and their whānau. Inclusion criteria for kaumātua research partners: self-identified Māori aged 60 + years with a self-reported and/or diagnosed hearing loss residing in Tāmaki Makaurau. Inclusion criteria for whānau members were: whakapapa whānau (members genealogically connected by common ancestors); and kaupapa whānau (members with a common purpose or disabling experience who provide supporting and nurturing roles that traditional whānau provide) (Metge 1995; Hickey and Wilson 2017). Potential kaumātua were invited to participate by a person known to them, e.g. community health worker, and provided with written and verbal information. The study was advertised at Waitangi day and marae events, Māori health and audiology clinics, and several social media, radio and website platforms. Kaumātua and their whānau who shared their interest in the TW project were contacted and provided with further information kanohi ki te kanohi (face-to-face).

Ten whānau interviews with twelve kaumātua and seven whānau members were performed between December 2019 and June 2020 (Table 1). Whānau interviews ranged from 45 minutes to 2 hours and were set at research partner homes and an aged-care centre. Kaumātua and whānau could select a pseudonym for confidentiality.

**Table 1.** Summary of kaumātua and whānau research partners.

Name of kaumātua research partner	Age Range	Degree and/or type of hearing loss (if known)	Has hearing technology	Whānau member/s interviewed
Adele	60–69	SNHL	Yes	–
Wai	80–89	SNHL	Yes	Daughter A Daughter B Daughter C
Rito	60–69	SNHL	No	Leonor
Riria	80–89	SNHL	No, previously did	Riria whānau member (does not wish to be named)
Julie	70–79	Not diagnosed	No	Toi and Dolly
Jeanette Patricia	70–79 70–79	SNHL SNHL	Yes Yes	Jeanette and Patricia are sisters and attend services together. No other whānau present
Nellsbell	70–79	N/A – childhood ear infection	No	–
Ruth Maraea	70–79 70–79	SNHL SNHL	Yes No, previously did	Ruth and Maraea are sisters, no other whānau present
Phyllis	80–89	SNHL	Yes	–
Rawiri	60–69	Mixed hearing loss	Yes	–

SNHL = Sensorineural hearing loss. Note: Some research partners used pseudonyms.

#### Focus groups

Focus group inclusion criteria were: HHCPs (audiologists, audiometrists, hearing therapists, and non-clinical HHCPs) and MHPs (medical and allied healthcare workers, community health and support workers who identified as Māori) working in Tāmaki Makaurau. For recruitment, the study was promoted kanohi ki te kanohi and advertised through newsletters and several organisation web-based platforms. HHCPs were recruited through NZ Audiological Society, audiology clinics and contacts, District Health Boards, and Life Unlimited Hearing Therapy. MHPs in Tāmaki Makaurau were contacted through marae, Māori Health organisations, General Practitioner (GP) practices, disability support services and allied health organisations/societies. HHCPs and MHPs were asked about their gender, occupation and what iwi (tribe/s) and ethnic group/s they identified with. All research partners were provided with an information sheet, consent form, and verbal information.

Two focus groups were undertaken (one with HHCPs and another with MHPs) online via Zoom in July 2020. Each focus group took approximately 1 hour and 40 minutes. The Zoom focus group meetings were recorded upon consent using the Zoom recording tool. In total, eight HHCPs (two hearing therapists and six audiologists) and six MHPs (pharmacist, kaumātua community support worker, hearing therapist, hearing-speech scientist/psychologist, two speech and language therapists) joined. One HHCP self-identified as Māori, and the remainder were non-Māori (one self-identified as Pacific). Among HHCPs, three worked in public HHC and the remainder privately. Key themes are reported for both focus groups combined and quotes detailed as HHCP1, HHCP2, MHP1, MHP2, etc. A Māori hearing therapist joined both focus groups (HHCP8 and MHP3).

Whānau interviews and focus groups held space for Māori cultural processes of: whakawhanaungatanga (the process of establishing and maintaining relationships); mihimihi (introduction and greetings); karakia (prayer/blessing); and whakawātea (reflections and clearing the pathway forward). Electronic data and audio recorded files were stored on a password-protected USB and deleted after transcription. At the appropriate COVID-19 response alert level in NZ, the lead researcher met with research partners to have a kōrero (discussion) on the study and share a koha (gift) including a supermarket/petrol voucher and a bag of kai (food). The koha and meeting in part reflects the research team's appreciation for the research partners’ time, wisdom, knowledge, and partnership. Several research partners asked to donate their koha, which was provided to a local marae in Tāmaki Makaurau.

### Data analysis

Audio recordings underwent transcription, translation, verification, and de-identification processes, with support from an external transcriber and certified ‘Te Taura Whiri i te reo Māori’ translator. Translated statements were reviewed by kaumātua and whānau research partners and transcribed data entered into NVivo 11 software. An inductive approach (Braun and Clarke 2013) was employed to identify patterns of meaningful insights across the dataset and allowed for kaumātua, whānau, HHCPs and MHPs to have phenomena explained from their own perspectives.

GS, EC, and ARM independently read and coded two transcripts (one whānau interview and one focus group transcript). This was followed with group discussions of overlaps and divergences in codes, and a final coding frame was decided. ARM coded remaining transcripts, and GS and EC provided feedback, allowing for inter-coding consistency. Māori researchers (EC and ARM) ensured the analysis and interpretation of data were appropriately aligned with KM principles. Codes were organised into appropriate themes through reflexive thematic analysis (Braun and Clarke 2013; Braun et al. 2019). Research partners were provided the option to share feedback to ensure accurate representation of their voices, and changes were made as requested. This process preserved the mana motuhake (independence, autonomy) and tino rangatiratanga (sovereignty, self-determination) of research partners.

## Results

Five main themes (hearing is a taonga for kaumātua, societal stigma of hearing loss, HHC barriers, whakawhanaungatanga in HHC, whānau-centred care and support) were identified and accompanied by 15 sub-themes (see Table 2).

**Table 2.** Themes and sub-themes: Whānau interviews and focus groups.

Main themes	Sub-themes
Hearing is a taonga for older Māori	Connecting through hearing and listening
	Managing hearing health to stay connected
Societal stigma of hearing loss	Impacts of colonisation on hard-of-hearing Māori
	Feeling whakamā to talk about ‘hearing loss’
	‘Living in a quiet world’ – Hearing loss is a struggle
HHC barriers	A Western non-Māori led HHC system
	Complex HHC processes and navigation system
	Socioeconomic, cost, location barriers
	Limited access to information on HHC and technology
Whakawhanaungatanga in HHC	Communication, connections and relationships
	Training Health Professionals
	Need for Māori HHC Workforce
Whānau-centred care and support	Support from whānau
	Whānau specialist support
	Supporting whānau of kaumātua

### Hearing is a taonga for kaumātua

Many kaumātua believe hearing is a taonga, a treasure to be valued and preserved. Narratives from whānau interviews and focus groups offer insight into why the sense of hearing is important and a taonga for Māori. This is discussed further in the following subthemes: (1) connecting through hearing and listening and (2) managing hearing health to stay connected.

#### Connecting through hearing and listening

Kaumātua have a deep appreciation for their sense of hearing. This notion is centred around their roles within whānau and communities, love for listening to te reo Māori (Māori language), hearing whaikōrero (formal speech), singing and listening to waiata (songs) at their marae and church, playing musical instruments, and for hearing their whānau especially their mokopuna (grandchildren). Gratitude for their sense of hearing to connect fostered a strong sense of belonging.

I love hearing te reo Māori, yeah. I love it. Well, it’s my heritage and I kōrero and whakarongo (listen). (Rito)

I realise it is an asset to hear. It’s your life really. Not just to be seen, but to be heard. (Maraea)

Several kaumātua found sounds to be ‘ihirangaranga’ (healing sound vibrations), especially when hearing te reo Māori or listening to other kaumātua speak.

Dolly: … if I'm not looking at the person and I can hear them, I can also feel what's coming from that language … it's the vibration of the certain words that they use. It has a wairua.

Julie: Ihirangaranga

HHCPs and MHPs also observed that Māori rely heavily on their senses to connect with people, the environment, their culture, and te Ao Māori (the Māori world).

#### Managing hearing health to stay connected

In the management of hearing health, kaumātua were willing to have regular hearing checks, use technology, take medication/s and use other strategies to remain involved in their communities. Hearing technology helped Ruth with playing piano, kept Wai and Phyllis safe, facilitated Jeanette and Patricia to hear on the phone better, assisted Adele and Rawiri with keeping focused in hui, and Maraea and Ruth noticed positive changes in whānau responses. Kaumātua however expressed issues with maintaining and managing their hearing aids, such as battery complexities and dexterity issues. Several kaumātua claimed that there was a need to be patient with oneself, and to stop and think about what their hearing loss and related symptoms such as tinnitus were telling them.

### Societal stigma of hearing loss

The second theme identified among all research partners was ‘societal stigma of hearing loss’. Many kaumātua shared intersectional experiences of being shamed and humiliated for being Māori and experiencing hearing loss at some stage in their life. These experiences impacted on how they perceived hearing loss and lived, dealt with, and sought out help for hearing loss. Several sub-themes encompassed this theme: impacts of colonisation on hard-of-hearing Māori, feeling whakamā (shy and ashamed) about hearing loss, and kaumātua ‘living in a quiet world’.

#### Impacts of colonisation on hard-of-hearing Māori

Many kaumātua shared challenging experiences of financial hardship, growing up in poor living conditions, and difficulties with migration to urbanised areas of NZ. Movement away from their whenua (lands) and support structures has had significant consequences on their identity, relationships, community connections, and financial and whānau support systems.

Her being in Auckland, I think that’s the restriction … if she was, in Gisborne or somewhere where a lot of things happened down there, I think she would be going. (Riria whānau member)

Kaumātua recalled being shamed and humiliated for having hearing difficulties, as well as traumatic experiences within an assimilated system that oppressed their identities as Māori and actively demeaned their culture and use of te reo Māori. As a hard-of-hearing tamariki Māori (Māori child) with middle ear issues in a native school, Rawiri said he ‘couldn't hear very well’, his teachers ‘felt that I didn't pay attention’ and ‘was strapped because he turituri ana [I was loud]’. However, ‘what they really didn't know is that I loved learning.’

#### Feeling whakamā to talk about ‘hearing loss’

Kaumātua spoke of feeling whakamā, shy, ashamed of their hearing loss with stigmatisation. They were labelled as old, and intellectually and mentally challenged because of hearing loss. Such stigma has influenced kaumātua decisions of accepting hearing loss, whether to be assessed and use of hearing technology.

I ignored it for a little bit cause I didn't want to be seen to be old and not you know not keeping up with everybody. (Adele)

Several MHPs observed kaumātua with hearing difficulties being judged and misdiagnosed.

Working in residential aged care, a few of the Māori people I saw with hearing loss that had never been diagnosed. So, people that had sort of been institutionalised their whole life because of what was thought to be psychiatric disorder but seems it was probably hearing loss and deafness. (MHP4)

#### ‘Living in a quiet world’ – hearing loss is a struggle

Research partners across all groups noted hearing loss as limiting, frustrating, isolating and tiring, with concerns that many are ‘living in a quiet world’.

When you see me switch-off, that means I’m not hearing you at all … I just go back to myself. (Riria)

HHCPs stated that to have a loss in more than one sense, one would require more processing, concentrating, listening effort, and consequently would get exhausted and fatigued. Kaumātua however believed that they were excluded from conversations because of the perception that they would get too exhausted in trying to keep up with conversations, resulting in not feeling valued.

They [people] get to the point where they don’t want to talk anymore. We thought, gee, maybe we’re not worth anything. (Maraea)

If you're not needed, or nobody wants you, then that’s a horrible feeling to feel, so it’s better to be over wanted than under wanted. (Wai)

### HHC barriers

A key theme from whānau interviews and focus groups was the experienced and perceived barriers to HHC for older Māori and their whānau. Several barriers to HHC were identified: Western non-Māori led HHC system, complex HHC processes and navigation system, socioeconomic barriers as well as cost and location barriers, and limited access to HHC and technology information.

#### A western non-Māori led HHC system

Negative healthcare experiences have generated and maintained distrust in the system, exacerbating health disparities. Several HHCPs suggest that a reason behind distrust is that the system is ‘too medically modelled’ (HHCP3). Several kaumātua felt ‘really strange’ (Rito) and ‘felt like an advertisement’ (Rawiri) rather than being provided the necessary assistance and tools for their hearing loss. Wai and her whānau said the ‘free hearing [check]’ was ‘like bait really’.

There are doubts among kaumātua and whānau about the effectiveness of a HHC system that lacks Māori leadership. In reforming HHC, kaumātua and whānau suggest partnering with ‘tangata whenua (people of the land)’ – Māori communities especially hard-of-hearing and d/Deaf Māori, kaumātua, marae leaders, church ministers, Māori providers and organisations, cultural advisors and government representatives.

#### Complex HHC processes and navigation system

Kaumātua reported going through long and complex processes of accessing HHC and long wait times for appointments. Riria required multiple appointments for earwax removal, which was difficult from a rest home. To get new hearing technology for Wai, daughter (C) reported, ‘It was probably nine months’. In some cases, clinics did not follow up with kaumātua.

Many kaumātua and their whānau are struggling to obtain administrative support to access HHC services and technology.

You have to have some language and engagement skills to be able to navigate some of the services … Then I think it's understanding what has been said to you. (MHP6)

MHPs therefore emphasised the importance of healthcare professionals in advocating for hard-of-hearing kaumātua and whānau, helping them navigate and access HHC and its processes effectively.

#### Socioeconomic, cost, location barriers

Several HHCPs indicated that the profession often overlooks socioeconomic status and the clients’ situation, background, needs and priorities.

They’re (healthcare system) not really considering the person’s life as a whole. (HHCP4)

Direct and indirect costs were discussed as barriers to accessing HHC services and technology. Kaumātua and whānau experienced difficulties in funding and assistance, where several applications were rejected. Kaumātua with occupational injuries, including head trauma and noise-induced hearing loss, did not meet the necessary criteria to obtain hearing technology funding through Accident Compensation Corporation (ACC). Narratives about Work and Income NZ (WINZ) assistance were generally positive, but there was criticism regarding WINZ complex processes and lack of user-friendliness.

Only a few kaumātua had insurance or applied for financial assistance, for example from a kaumātua trust. Those without insurance or financial assistance struggled with the cost of replacing their hearing technology. HHCPs voiced concerns of growing inequities and highlighted the need to revamp HHC funding systems.

Most kaumātua and their whānau preferred General Practitioners (GPs) as their first point of contact for hearing challenges and emphasised the importance of community-based services. In some cases, HHCPs and MHPs discussed several policies that restricted their ability to refer or recommend beneficial community-based organisations for kaumātua. HHCPs also discussed that whilst they ‘should be more accommodating,’ they should also be asking critical questions around inequality and inequity.

#### Limited access to information on HHC and technology

A common account reported was the lack of understandable and transparent information about services, hearing loss, hearing technology (e.g. devices and Bluetooth), costs, ethnic-specific data, and hearing health promotion, without use of jargon/abbreviations/acronyms.

No one here [rest home] knew what an ORL was. (Riria whānau member)

Several HHCPs believe Māori prefer the simplest of HHC solutions. In contrast, some HHCPs discussed providing kaumātua with all available options. HHCPs and MHPs highlighted the need to connect with other health professionals to understand what services are available to help their kaumātua clients and whānau. Additionally, kaumātua and MHPs discussed the need for hearing health promotion.

The absence of data on identified hearing loss prevalence among kaumātua affects HHC delivery, with HHCPs concerned that private clinics are not collecting ethnicity information or making it mandatory. When asked why ethnicity data was not collected, research partners said it was a ‘cheat to the system’ (HHCP6) and ‘you’d have to ask our directors’ (HHCP1). Several HHCPs found this problematic.

If somebody walked in, they wouldn’t know if they were Tongan or Samoan or any of that, and I never hear anybody ask. (HHCP4)

### Whakawhanaungatanga in HHC

A common thread amongst all groups was the importance of ‘Whakawhanaungatanga in HHC’. This theme emphasises the importance of building connections and relationships between kaumātua, whānau and their HHCPs. The sub-themes tied to this were: communication, connections, relationships; training health professionals; and need for Māori HHC workforce.

#### Communication, connections, relationships

Kaumātua had positive experiences of HHC when supported and provided with the tools to manage hearing difficulties and related symptoms by HHC staff and/or external healthcare workforce (e.g. community support workers). In Julie’s words, ‘they include me in their discussions.’

When audiologists effectively used their time to understand kaumātua and their whānau experiences and explain everything clearly, many kaumātua felt heard and accommodated. In the absence of whakawhanaungatanga, kaumātua and their whānau have felt disconnected and unwelcome, particularly when appointments were focused on problems of the hearing system rather than their communication experiences and needs.

There’s a way and a way of talking to people … they don’t know how you do that. (Riria)

I was having very bad tinnitus, and she gave me some sort of hearing aid that was supposed to do something with the tinnitus. I wore it, and it did feel as though it helped me lots, and then I had to send it into repairs, and they said that I’d missed the date. So, I didn’t bother going back … (Jeanette)

MHPs and HHCPs observed health professionals blaming Māori for not attending services. HHCP2 reflected on getting ‘sucked into that mindset. I’m sitting there going, oh, another DNA blah, blah, blah.’

The idea they wouldn’t come because they don’t care is just so ignorant and upsetting. (MHP2)

Embedding Māori values like whanaungatanga and manaakitanga (act of support and showing respect) and interweaving lived experiences within that are imperative. Rawiri called this ‘transformative consultations’ and is beneficial within many services and for many other communities.

I basically treat them like they're my own nanny and koro. (MHP1)

#### Training health professionals

Several HHCPs believed that to change and shift practice they too need to strengthen connections with their clients and be ‘voices for change as well’ (HHCP2). Some HHCPs said that to build trust, improve client care and reduce client anxiety, they made time to ask relatable questions and share their own experiences in the community. HHCP8 shared her experience as a hard-of-hearing wahine Māori with hearing technology which opened opportunities to connect with Māori clients’ and reduce stigma of hearing loss and hearing technology.

Moreover, several kaumātua and HHCPs noted proper pronunciation of names and te reo Māori use in HHC play a part in connections and positive experiences with kaumātua and whānau.

Te reo was his (child patient’s) first language … I sort of had to do it (assessment) ad hoc with Dad’s help, and it worked. (HCCP2)

When you work in Whāngarei, as we say to the new graduates, there is going to come a time when you’re going to have to do the Kendall Toy Test in Māori because that is going to be the child’s first language. (HHCP1)

Several kaumātua mentioned that other than mihimihi and pronunciation, HHCPs’ training should involve building the skills to be sensitive to their clients, understanding of client experiences, and being culturally safe, so Māori are well-informed and have the knowledge and agency to make the best choices. HHCPs noted the limited Te Tiriti o Waitangi (Te Tiriti), Indigenous health, and cultural support training they get through tertiary HHC courses and their professional career. MHPs and HHCPs discussed the need to review their ways of thinking and practices for the benefit of hard-of-hearing communities and their whānau.

Reviewing our own thinking and our own practice and just really thinking about how we look at the stuff that isn’t easily testable. The stuff that people don’t know to talk about. (MHP6)

#### Need for Māori HHC workforce

Kaumātua stated their whānau may not always be around to attend appointments and not all kaumātua feel confident advocating for themselves in healthcare. Kaumātua mentioned the limited expectations of seeing Māori HHCPs, and highlighted the want for more, particularly with kaumātua feeling comfortable to share experiences ‘Māori-ki-te-Māori’ (Māori-to-Māori). Several kaumātua recommended investing in Māori and Pacific community support workers, facilitators, navigators, carers due to valued support they provide at home and in accessing services.

Knowing you’re Māori makes it more comfortable, less whakamā about what we have to say. (Maraea)

HHCPs acknowledged the connections, trust and comfort that Māori clients’ have with Māori and Pasifika staff.

My front-of-house, she’s Māori and I was just thinking, I don’t really seem to see too many barriers when people are coming in. They seem to be fairly relaxed and maybe because she’s an icebreaker. (HHCP6)

The Māori woman came in, and she saw me at the door, and she said to her own daughter, oh look, there’s a Māori girl. (HHCP8)

MHPs suggested Māori are more likely to take up a particular profession if they have personal or whānau experiences relating to that field or see Māori in that profession. MHPs considered the education challenges of accessing technology, resources, and funding and Māori HHC workforce growth. MHPs further commented on the ‘struggle to practice as Māori’ (MHP5), where Māori are expected to do things that are beyond their scope of practice and what they are paid to do. With limited support in their respective health fields there is hesitancy in promoting their profession, emphasising the need to support Māori allied health students and workforce.

It becomes your responsibility to teach non-Māori clinicians about what to do. But are you being provided with the assistance to figure that out yourself? (MHP6)

### Whānau-centred care and support

The final theme concerns the important support roles of whānau in navigating HHC and the need to support whānau-centred HHC. Three sub-themes are acknowledged and will be discussed: support from whānau, whānau specialist support, and supporting whānau of kaumātua.

#### Support from whānau

Kaumātua referred to intergenerational whānau support. Several kaumātua reminisced about their own elders experiencing hearing loss and having to assist them on the paepae (place on the marae where orators sit, stand, and deliver their speeches). As years passed, kaumātua find themselves in similar situations and appreciate their whānau for helping them communicate. Riria said, ‘I find shopping really hard. You know, you’ve got to find someone to have the patience enough too – to go and help.’ Riria’s whānau member assists most of the time, ‘I always have to, you know, interpret or speak to them (shop assistants)’. In another example, Ruth assists Maraea with communicating in appointments. Maraea said, ‘She hears better than me … That’s why I ask her’. HHCPs observed this support in their clinics.

#### Whānau specialist support

Kaumātua perspectives of whānau specialist support to navigate services, understand their hearing loss, assist with funding applications and upkeep of hearing technology are presented alongside support from whānau members.

If I wasn’t able to go and pick up the batteries, I could always get my son to call in and pick them up. Or my daughter on her way back from work. (Phyllis)

Sisters, Patricia and Jeanette, relied on each other for further information about their hearing technology:

Jeanette: I’ll tell you what I learnt from seeing [de-identified], the audiologist, two weeks ago, is that Patricia that you’ve got to get that little like a needle thing … you’ve got to get that to get some wax out because you mightn’t see it visually but if you get your glasses on, even the slightest bit of wax … 

Patricia: Where do you put the needle? See, I wasn’t told any of that, they just said, this is it.

#### Supporting whānau of kaumātua

Whānau-centred care is ‘not interfering with the service’ (Julie). MHPs stated that whānau of kaumātua were often advocates for their elders’ hearing health and wellbeing and wanted to be part of the processes in HHC. MHPs saw that when whānau members of hard-of-hearing kaumātua were provided with the right guidance, tools, and sources, they become more confident in supporting their own whānau.

Whilst whānau want to be part of HHC journeys, many HHC services that kaumātua encountered had an individualised, patient-centred care approach. Involving whānau in HHC is however required to understand third-party challenges and provide awareness and education on hearing health and communication within whānau and communities. HHCPs also mentioned they can attend to the communication needs of both the patient and their whānau.

Maybe to understand what’s her best level of hearing? (Riria whānau member)

It's an educational process for family as well to be involved. (MHP1)

From our perspective to be able to really get a feel for what-where the communication is difficult. (HHCP7)

## Discussion

The key findings from whānau interviews and focus groups showed that hearing is important and a taonga for older Māori. However, colonisation, societal stigma of hearing loss, and barriers to accessing HHC and hearing technology continue to impact older Māori with hearing loss. The TW study further showed the importance of whakawhanaungatanga and the need for whānau-centred HHC. These findings provide a foundation for three discussion themes accompanied by recommendations: (1) ‘me rongo i te whanaungatanga’ (to feel connected); (2) ripple effects on hard-of-hearing kaumātua of colonisation, HHC barriers and stigma; (3) ‘au, whānau, whakawhanaungatanga’ – shift to whānau-centred care.

### Me rongo i te whanaungatanga – to feel connected

Growing older for Māori is an intergenerational responsibility where strengthening and maintaining quality relationships with their whānau and environment/s are of high priority. In this study, kaumātua discussed the need to have their hearing to engage with their whānau, iwi, and hapū, as well as (re)connect with their whakapapa (genealogical relation to all things living and non-living) and Te Taiao (the natural world). Similarly, Walsh (2020) found a Gunggandji Traditional Owner shared the value of his hearing, especially out in the country to hear nature, the birds, wildlife, wind and water running. Furthermore, several kaumātua acknowledged specific sounds as ‘ihirangaranga’ – sound vibrations that continue to carry and impart source vitalities (Rostenburg 2020, p. 90). As an example, karanga (ceremonial call or summoning) can be felt through the energy of its vibrations and the weaving of spiritual power within particular sounds (Browne 2005; Pere 2012; Rostenburg 2020). Meyer (2001) noted Hawaiian people interpret listening as a spiritual act, because to ho’olono (listen) is to invoke a spirit, a deity, and is tied to whakapapa and learning of what is most critical from Indigenous worldviews.

Listening is important for preserving Indigenous methods of knowledge sharing, language, culture, whakapapa, and wellbeing. Within te Ao Māori, learning and passing of knowledge is highly dependent on physiological senses (e.g. listening for a birds warning) to observe, listen, stay attentive and retain information (Wikaire 2020). Miriam-Rose Ungunmerr-Baumann – a Ngangikurungkurr ‘the Deep Water Sounds’ elder (Daly River, Northern Territory) – noted that for over 40,000 years Aboriginal peoples practiced ‘dadirri’, a practice of deep listening, quiet stillness and respect (Ungunmerr-Baumann and Brennan 1989). She writes,

Through the years, we have listened to our stories. They are told and sung, over and over, as the seasons go by. Today we still gather around the campfires and together we hear the sacred stories. As we grow older, we ourselves become the storytellers. We pass on to the young ones all they must know. (p. 40)

In connection to this, the art of listening among the Sweetgrass nation in Saskatchewan has been described as a way of life deeply rooted in traditions and teachings (Netmaker 2023). The bowing of heads and avoiding eye contact represented a state of focus and learning and are gestures of respect towards elders and community leaders who impart wisdom and stories.

### Ripple effects on hard-of-hearing kaumātua of colonisation, HHC barriers and stigma

In this study, hard-of-hearing kaumātua shared the impacts of colonisation, assimilation, and migration from their whenua. Many of whom have encountered marginalising and intersectional experiences of racism, ageism, ableism and disablism. This is resonated in other research that shows Indigenous peoples with disabilities, including those who suffer from hearing loss, often experience a triple disadvantage due to having a disability, being Indigenous, and residing in areas remote from service provision centres (Gething 1995; Maher 1999; Nikora et al. 2004).

Kaumātua and whānau in this study recalled the unaffordability of HHC services and technology. They also noted limited opportunities for whānau to attend and/or participate in HHC appointments, difficulties in navigating the HHC system and its funding policies, inadequate information and options provided by their HHCPs, and poor access to follow-up, review, maintenance or support services. These ear and hearing healthcare barriers for Māori resemble those reported in the NZ Human Rights Commission report (2020) and other ear and hearing health Māori-led research (Buckthought et al. 2024; Crisp 2010; Dargaville 2010; Lowe 2023; Williams 2019).

Similar to findings of Williams (2019) and Perkins and Coombes (2006), kaumātua particularly those without hearing technology have been stigmatised for having hearing loss, felt isolated, withdrawn from activities/events and had difficulties with (re)learning te reo Māori. As a result, kaumātua are finding themselves living in a silent world, which may impact kaumātua taha hinengaro (mental and emotional wellbeing), one of several important dimensions of hauora Māori (Māori health) (Durie 1984). Consequences of middle ear issues and hearing loss among Indigenous peoples have contributed indirectly to the linguistic and cultural barriers that constrain communication (Howard 2007) and are connected to depression for Indigenous peoples (de Plevitz 2010).

### ‘Au, whānau, whanaungatanga, whakawhanaungatanga’ – shift to whānau-centred care

Whānau, as the core social unit, play a crucial role in supporting kaumātua with hearing loss, aiding in communication, daily activities, and managing listening devices at home. Likewise, Durie (1999) and Dyall and colleagues (2011) reported many kaumātua could rely on whānau assistance with financial aid, accommodation, communication in te reo Māori, and transport and support when unwell. Whānau involvement in appointments is however often overlooked, unpaid, and potentially devalued if not recognised (Graham and Masters-Awatere 2020). This supports the need to centralise whānau-centred care – valuing whānau as a whole, building on whānau strengths and increasing their capacity (Durie et al. 2010).

The significance of relational approaches, specifically whanaungatanga, appears crucial in growing and strengthening trust of kaumātua and their whānau in HHC. Māori scholars, Burgess and Painting (2020), explain the web of whanaungatanga as being in good relation, where ‘whanaunga’ implies relation and ‘tanga’ refers to being in the state of: To be in good relation is our connections with our cultures, worldviews, times, languages, histories, spiritualities, and places in the cosmos (Wilson 2008). As such, whakawhanaungatanga goes beyond building rapport. It is the act of long-term relationship-building processes, expanding Māori HHC leadership and workforce through pipeline or pathway models (Curtis et al. 2012; Ratima et al. 2007), and communicating and conversing about shared commonalities and experiences (Lacey et al. 2011; Wepa 2016).

Research partners also called for cultural safety to be implemented. By embedding culturally safe praxis healthcare professionals can recognise barriers to healthcare that stem from power imbalances between providers and clients (Curtis et al. 2019). Pillay and Kathard (2015) further suggest ‘critical consciousness’ among HHC professionals should be fostered as a start to shifting the locus of power and control to clients.

The research and praxis behind whānau-centred care in HHC remains limited. Substantial international audiology research supports the move towards family-centred care (FCC), where both the person with hearing loss and those around them should be the focus of audiologic rehabilitation (Ekberg et al. 2015; Hickson et al. 2016; Ekberg et al. 2020; Maluleke et al. 2021; Nickbakht et al. 2021; Scarinci et al. 2022; Ekberg et al. 2023). For whānau-centred care, Smiler (2014) proposed that supports and services for Māori deaf children and their whānau should be arranged within the concept of ‘pā harakeke’ (harakeke [flax] plantation). The pā harakeke represents the competency of whānau and is likened to the collective community and whānau protecting the rito (the shoot or child) or the kōhungahunga turi (young deaf person). Given the diverse nature of family and whānau networks structurally and functionally, there is a need for further exploration of family – and whānau-centered HHC in NZ.

As these are further explored Tāngata whaikaha kaumātua (kaumātua with lived experiences of disability) and their whānau should be involved in decision-making structures that impact their health and wellbeing (Ingham et al. 2022). Under Te Tiriti o Waitangi, the NZ Human Rights Act (Ministry of Justice 1993), and United Nations Declarations and Conventions (United Nations 1965, 1966, 2006, 2007), hard-of-hearing kaumātua and their whānau have the right to health and wellbeing, the right to access HHC that is free of discrimination, as well as the legal right to choose, determine and govern their own health and wellbeing. Shifts towards Māori paradigms and frameworks that take upon a decolonial stance will assist in centralising kaumātua concerns and worldviews for Māori purposes and provide space to question systems and models of care that maintain the status quo (Smith 2012).

### Research directions

More qualitative KM research is needed to understand Māori experiences and perspectives on Māori models of ear and hearing health, ear/hearing-related conditions, improving HHC workforce pathways, connections through senses and addressing stigma of hearing loss and technology, and culturally responsive whānau-centred HHC delivery and provision. KM approaches to quantitative methods of research could also be beneficial for example in identifying ethnic-specific prevalence and causes of hearing loss in NZ (e.g. noise-induced hearing loss), and who is applying and approved for funding of HHC services and technology. Overall, KM mixed-methods research would assist in understanding the state of hearing loss and HHC in NZ and strengthen arguments to fund equitable and responsive policies, services, initiatives, technology that advance Māori hearing health outcomes.

### Strengths and limitations

A KM approach was supported with feedback from Māori community organisations, rōpū (groups), and advisory board members, ensuring safety, responsiveness, and relevance for all research partners involved. Notably, the application of KM research marks the pioneering use of a strengths-based application in HHC research to highlight research partners’ expertise and significance throughout this study. A research partner took part in both HHCP and MHP focus groups, this could be viewed as a limitation of the study given the potential of group bias and possible crossover of knowledge or contamination of ideas from one focus group to another. The labelling of their contributions as HHCP8 and MHP3 mitigates this concern.

## Conclusion

The TW project captures the lived experiences and perspectives of hearing loss and HHC among older Māori and whānau residing in Tāmaki Makaurau. It also shares HHCP and MHP perspectives on hearing loss and HHC provision. Research partner narratives drew attention to the need for transformation of HHC that is self-determined by Māori and their whānau to advance Māori health and wellbeing. It is hoped that the information gathered from this study will spark further ideas and opportunities around the provision of HHC that are by, for, and with Indigenous elders and families worldwide.
